# Sub-15-nm patterning of asymmetric metal electrodes and devices by adhesion lithography

**DOI:** 10.1038/ncomms4933

**Published:** 2014-05-27

**Authors:** David J. Beesley, James Semple, Lethy Krishnan Jagadamma, Aram Amassian, Martyn A. McLachlan, Thomas D. Anthopoulos, John C. deMello

**Affiliations:** 1 Department of Physics, Imperial College London, Prince Consort Road, South Kensington, London SW7 2AZ, UK; 2 Department of Chemistry, Imperial College London, Exhibition Road, South Kensington, London SW7 2AZ, UK; 3 Division of Physical Sciences and Engineering, Solar and Photovoltaic Engineering Research, King Abdullah University of Science and Technology (KAUST), Thuwal 23955-6900, Saudi Arabia; 4 Department of Materials, Imperial College London, Exhibition Road, South Kensington, London SW7 2AZ, UK; 5 These authors contributed equally to this work

## Abstract

Coplanar electrodes formed from asymmetric metals separated on the nanometre length scale are essential elements of nanoscale photonic and electronic devices. Existing fabrication methods typically involve electron-beam lithography—a technique that enables high fidelity patterning but suffers from significant limitations in terms of low throughput, poor scalability to large areas and restrictive choice of substrate and electrode materials. Here, we describe a versatile method for the rapid fabrication of asymmetric nanogap electrodes that exploits the ability of selected self-assembled monolayers to attach conformally to a prepatterned metal layer and thereby weaken adhesion to a subsequently deposited metal film. The method may be carried out under ambient conditions using simple equipment and a minimum of processing steps, enabling the rapid fabrication of nanogap electrodes and optoelectronic devices with aspect ratios in excess of 100,000.

## Abstract (web summary)

Electron-beam lithography is often used for patterning of asymmetric metal electrodes for nanoscale devices, but suffers from several limitations. Here, the authors report a new adhesion lithography process, which allows for high-throughput and simple fabrication of nanogap metal electrodes.

## Graphical abstract

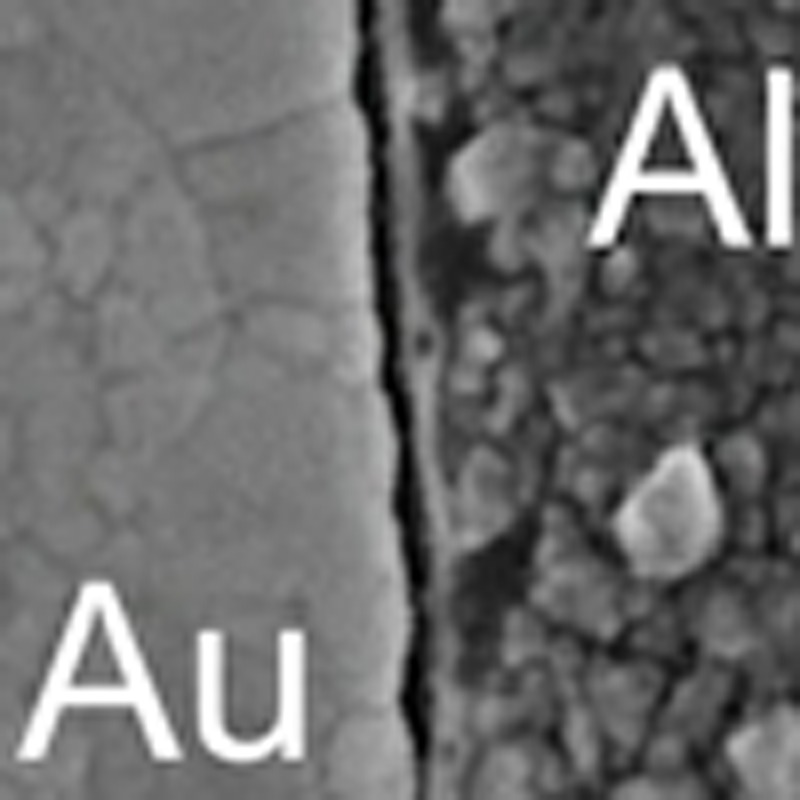

Laterally aligned metal electrodes, separated on the nanometre length scale, are essential elements of many nanoscale photonic and electronic devices^1^, offering key advantages over sandwich structures in terms of higher fabrication yields, greater amenability to large-scale integration, reduced parasitic capacitances and lower leakage currents^2^. Existing fabrication routes entail the use of electron-beam lithography^3^,^4^,^5^,^6^, mechanical break junctions^7^,^8^, electrochemical deposition^9^,^10^,^11^, oblique-angle shadow-evaporation^12^, scanning probe lithography^13^ or on-wire lithography^14^ to achieve intimate registration of the two electrodes. Such methods, however, variously suffer from low throughput, poor scalability to larger substrate sizes, complex multi-step processing protocols, and/or high equipment costs^1^,^15^. In addition, most techniques are limited to the patterning of a single materials system, and consequently cannot be applied to the fabrication of asymmetric nanoscale devices such as rectifiers and ambipolar devices that require the use of closely spaced dissimilar metal electrodes.

Here, we report a method for fabricating arrays of high aspect ratio asymmetric nanogap electrodes, exploiting the ability of selected self-assembled monolayers (SAMs) to attach conformally to a prepatterned metal layer and thereby weaken adhesion to a subsequently deposited metal film. The method—which can be carried out at room temperature under ambient conditions, using simple equipment and a minimum of processing steps—provides a rapid route to highly aligned, electrically isolated, asymmetric electrodes separated on the nanometre length scale.

## Results

### Description of patterning procedure

**Figure 1:**
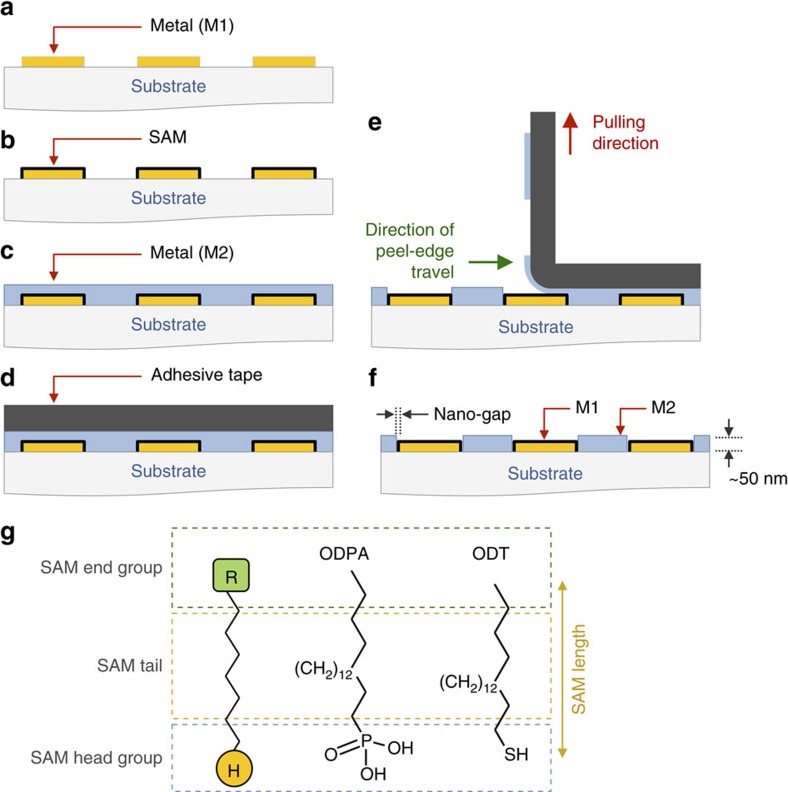
Schematic showing the principle of adhesion lithography and suitable self-assembled monolayers (SAMs). The patterning procedure comprises the following steps: first, metal M1 is deposited on a substrate (**a**); second, M1 is selectively coated with a metallophilic SAM (**b**); third, metal M2 is deposited uniformly over M1 and the exposed substrate (**c**); fourth, adhesive tape—or an alternative adhesive material—is applied to the surface of M2 (**d**); and, finally, the tape is peeled away from the substrate, selectively removing M2 from those regions located directly above the SAM (**e**). On completion of the patterning procedure, M1 and M2 sit side-by-side on the substrate in a complementary arrangement (**f**), separated in the limiting case by the length of the SAM. For patterning, the SAMs require a metallophilic head group and a metallophobic alkyl tail: octadecanethiol (ODT) was used for attachment to gold, while octadecylphosphonic acid (ODPA) was used for attachment to oxide-coated aluminium (**g**).

The patterning method employed here—termed adhesion lithography (a-lith)—(Fig. 1a–f) entails deposition onto a substrate of a thin (~\n50 nm) metal film (M1), which is selectively patterned to expose the underlying substrate in regions where a second metal is later to be deposited (Fig. 1a). An alkyl-terminated metallophilic SAM (see Fig. 1g) is conformally attached to all exposed surfaces of the metal, with the alkyl chains facing outwards from the metal surface (Fig. 1b). Next, a second metal film (M2) is uniformly deposited over the full area of the substrate (Fig. 1c). Owing to the presence of the SAM, the adhesion of the second metal to M1 is much weaker than its adhesion to the substrate. In consequence if adhesive tape—or an alternative adhesive material—is applied uniformly to the surface of M2 (Fig. 1d) and then peeled away (Fig. 1e), M2 will detach from the regions above M1 and remain only in those areas where the substrate was previously exposed. Hence, at the end of the procedure the two metals will sit in a complementary arrangement side-by-side on the substrate, separated in the limiting case by just the length of the SAM—a few nanometres or less (Fig. 1f). We note in passing that the conformal attachment of SAMs to a prepatterned metal has previously been exploited to define arrays of nanostructures with tightly controlled nanoscale spacings. In that case, however, the SAMs were used as an evaporative shadow mask, with nanoscale voids between the SAMs providing selective access to the underlying substrate^16^.

M1 and M2 may be identical or dissimilar metals according to need but for ease of imaging all results presented here involve the use of dissimilar metals, namely gold and aluminium—the latter coated with a native surface layer of oxide (alumina). For successful patterning, the adhesion between the adhesive tape and M2 must be weaker than the adhesion between M2 and the substrate but stronger than the adhesion between M2 and the SAM, which can be ensured by judicious selection of the adhesive tape and SAM. In circumstances where the adhesion of M1 or M2 to the underlying substrate is too weak, the substrate must be coated with an adhesion-promoting layer before metal deposition. When gold is used for M1 (and aluminium for M2), it is sufficient to coat the substrate with a thin (~\n5 nm) layer of evaporated chromium. When gold is used for M2 (and aluminium for M1), however, we have found it beneficial to coat the substrate with a 2 μm layer of the thermally curable resin bisbenzocyclobutene (BCB)^17^ to ensure adequate adhesion of gold to the substrate (see Methods).

The appropriate selection of SAM is critical for achieving reliable patterning. Here, we used SAMs of octadecanethiol (ODT) for attaching to gold and octadecylphosphonic acid (ODPA) for attaching to slightly oxidized aluminium (see Fig. 1g and Methods), both having been successfully deployed as ultrathin dielectrics for organic transistors^18^,^19^.

### Modifying adhesion strength with SAMs

**Figure 2:**
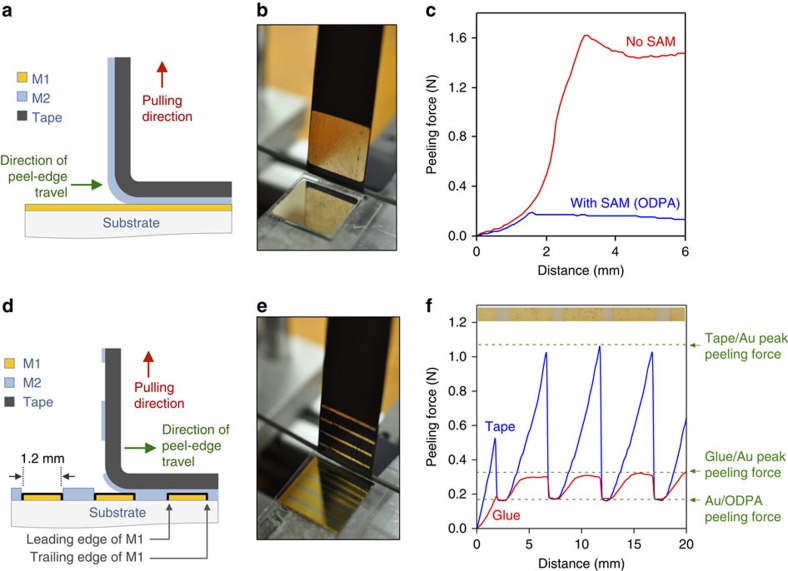
Peel test measurements for Au on Al. (**a**) Schematic of 90° peel test applied to a layer of Au on top of a uniform layer of ODPA-coated Al. (**b**) Photograph showing the tape after peeling, with removed Au visible on the tape. (**c**) Peel curves for a layer of Au on top of a uniform layer of Al, with and without an intervening ODPA layer. Without ODPA, the applied force peaks at a high value of ~\n1.6 N, before decreasing to a lower steady state value of ~\n1.4 N. With ODPA, the applied force peaks at a much lower value of~\n0.2 N before falling to a steady state value of~\n0.16 N. (**d**) Schematic of 90° peel test as applied to a uniform layer of Au deposited on a line array of ODPA-coated Al with spacings, widths and lengths of 4, 1.2 and 20 mm, respectively. (**e**) Photograph showing the tape after peeling, with removed gold visible on the tape. (**f**) Peel curves for Au films on top of ODPA-coated Al line arrays, using insulation tape or solution-coated glue as the adhesive layer. In the case of the tape, a linear increase in the peeling force from ~\n0.16 N to ~\n1 N is observed as the peel edge passes across the (Al-free) regions between lines. This is followed by a rapid decrease to ~\n0.16 N as the peel edge passes onto the ODPA-coated Al, and the tension drops due to the weakened adhesion. In the case of the glue, a similar peeling force of ~\n0.16 N is measured when the peel edge is above the Al lines but the behaviour in-between lines is different, with the peeling force stabilizing rapidly to ~\n0.35 N. Inset is a photograph of the final patterned structure. See Supplementary Note 1 for a discussion of all four peeling curves.

To investigate the influence of the SAM on the adhesion between the two metals, a 40 nm uniform layer of Al (M1) was deposited onto 2 × 2 cm BCB-coated glass, followed by a monolayer of ODPA and a 40 nm uniform layer of Au (M2). Control structures were fabricated by omitting the SAM and directly depositing Au on top of Al. The force required to remove the Au from the ODPA-coated Al was then determined by peel testing^20^, in which the former is controllably peeled from the latter and the associated peeling force recorded using a strain gauge (Supplementary Fig. 1). To initiate peeling, highly adhesive electrical insulation tape was applied evenly across the surface of the Au and peeled back at a constant rate of 10 mm s^−1^, 90° to the surface (see Methods and Fig. 2a). The presence of the ODPA monolayer enabled clear separation of the two metal films, with the top layer of metal detaching easily from the SAM and transferring to the adhesive tape with minimal residue on the substrate (Fig. 2b). The peak peeling force was reduced 10-fold from ~\n1.6 N without the ODPA monolayer to ~\n0.16 N with (Fig. 2c). Equivalent measurements using Al (M2) on top of Au (M1) yielded broadly similar results, with the peak peeling force being reduced from ~\n1.6 N without the ODT monolayer to ~\n0.3 N with (see Supplementary Fig. 2). The efficacy of the SAM layers in reducing the adhesion between the upper and lower metals was further confirmed by adhesion force microscopy, which for both Au on ODPA-coated Al and Al on ODT-coated Au showed a >fivefold reduction in the adhesive forces relative to the SAM-free case in broad agreement with the peel test data (see Supplementary Fig. 3).

### Adhesion characteristics of line arrays

For initial low-resolution patterning tests, line patterns of Al (M1) were deposited onto BCB-coated glass (see Fig. 2d), a SAM of ODPA was applied to the Al and a uniform layer of gold (M2) was then deposited across the full area of the substrate. Adhesive tape was applied uniformly to the surface of the Au and then pulled away from the substrate at 90° using the peel tester (*V*=10 mm s^−1^), with the peel edge travelling in a direction perpendicular to the Al lines (see Methods and Fig. 2d,e). The strain gauge registered a low value of <0.2 N for the applied force whenever the peel edge was located directly above one of the SAM-coated lines of Al and recorded a steady linear increase to ~\n1 N whenever the peel edge passed beyond M1 into the region between lines, before falling back to <0.2 N when the peel edge reached the next Al line (see blue line in Fig. 2f). Reversing the structure—using Au on Cr-coated glass for the line pattern (M1), ODT as the SAM, and Al as the top layer of metal (M2)—yielded broadly similar results, with the strain gauge registering a low value of ~\n0.25 N whenever the peel edge was located directly above the Au line pattern (M1) and increasing approximately linearly to a peak peeling force of ~\n1.1 N when the peel edge was located between lines (see Supplementary Fig. 4). In both cases, the behaviour was consistent with peeling occurring at the weakly adhered SAM/M2 interface whenever the peel edge was located above M1 and occurring at the strongly adhered M2/tape interface otherwise. This interpretation was confirmed visually by inspecting the underside of the tape after peeling, which showed lines of peeled M2 that mirrored the original line pattern of M1 (see Fig. 2e and Supplementary Fig. 4a).

### Influence and selection of the adherent

In practice, owing to its stiff texture, the insulation tape cannot make truly conformal contact with the underlying surface, and consequently it is not always possible to completely remove the unwanted parts of M2 in a single peeling step, as is evident from the imperfect line profiles in Fig. 2e and Supplementary Fig. 4a. In these circumstances, a second (or occasionally third) peeling step is required to remove the residual material. To avoid the need for multiple peeling steps, the insulation tape was replaced by a solution-deposited glue (First Contact, Photonic Cleaning Technologies) that, on drying, forms a thin-film polymeric coating in intimate contact with the underlying surface. The effect on the peeling forces of switching from the adhesive tape to the solution-deposited glue is shown in Fig. 2f, which depicts peel curves for gold films on top of ODPA-coated Al line arrays, using insulation tape or solution-coated glue as the adhesive layer. (The different profiles of the two peel curves—a regular sawtooth in the case of the tape versus a periodic rise/plateau/drop in the case of the glue—are discussed in Supplementary Note 1). The peel force differential between the leading and trailing edges is much smaller in the case of the solution-deposited glue, leading to a more controlled peel as discussed below. For the avoidance of doubt, the term leading edge refers to the edge of M1 that is encountered when the peel edge passes from M2 to M1 and the term trailing edge refers to the edge of M1 encountered when the peel edge passes from M1 back to M2, see Fig. 2d.

The fidelity of the patterning process depends on several factors including: the topographies of M1 and M2; the relative adhesive forces between the various layers; the direction of peeling; and the tackiness and elasticity of the adhesive layer. For complete and accurate patterning, M2 must fracture sharply at the leading edge of M1 (allowing M2 to detach from the SAM), peel in a continuous strip until the trailing edge of M1 is reached and then fracture sharply again (allowing M2 to remain attached to the substrate). The forces generated at the leading and trailing edges of the line pattern—which in general can differ significantly—have a strong influence on the patterning resolution. In particular, excessively strong peeling forces on approaching the leading edge can cause premature fracturing of M2 on the substrate, while excessively weak peeling forces on leaving the trailing edge can cause delayed or failed fracturing of M2 on the tape—both cases resulting in deterioration of the patterning resolution. While the glue and adhesive tape provided similar forces of <0.2 N on leaving the trailing edge, the glue provided a much smaller force *F* (and slope d*F*/d*x*) on approaching the leading edge of ~\n0.35 N (compared with ~\n1.1 N for the adhesive tape), making it the more appropriate choice when high-resolution patterning is required. Using adhesive tape, we have found it difficult to achieve electrode spacings below 100–200 nm (see Supplementary Fig. 5). Using the solution-processed glue, by contrast, electrode spacings of just a few tens of nanometres can be realized as we describe below.

### High-resolution patterning using solution-processed glue

**Figure 3:**
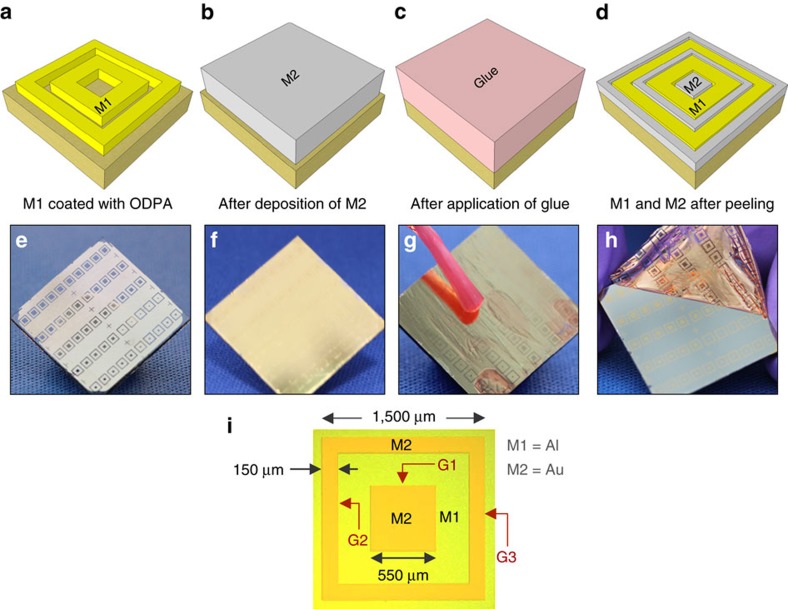
Fabrication of concentric square electrodes. (**a**–**d**) Schematic illustrating procedure for the fabrication of aligned concentric squares of Al and Au with a central square of Au, involving: first, deposition of Al (M1) in a ‘square-ring in square-ring’ geometry, followed by treatment with ODPA; second, deposition of a uniform layer of Au (M2) on top of the patterned Al and the exposed substrate; third, application of solution-deposited glue across the entire surface of the Au; and, fourth, removal of the glue to peel away unwanted parts of Au (that is, those lying above Al), leaving behind an in-plane concentric arrangement of Al and Au. (**e**–**h**) Photographs of the same four processing steps; substrate size is 20 × 20 mm. (**i**) Micrograph of resultant concentric square pattern; G1, G2 and G3 denote gaps between the two metals.

To demonstrate the viability of using a-lith to pattern extremely close-packed geometric features, we sought to fabricate alternating concentric patterns of dissimilar metals (Au and Al), with the objective of achieving nanoscale separations between the two metals—a challenging task that is difficult to achieve using conventional patterning methods. To prepare structures with a central square of gold, patterned aluminium (M1) was first deposited on BCB-coated glass in a concentric ‘square-ring in square-ring’ arrangement using standard low-resolution etching (see Methods and Fig. 3a). Next, a monolayer of ODPA was applied to the aluminium, and a uniform layer of gold (M2) was deposited across the full area of the substrate (Fig. 3b). The glue was then applied uniformly to the surface of the gold and allowed to dry (Fig. 3c). Finally the glue was peeled away, selectively removing those parts of the gold that lay directly above the aluminium and leaving behind an alternating, concentric arrangement of the two metals with an inner square of gold (Fig. 3d). Photographs at each stage in the fabrication procedure are shown in Fig. 3e–h, while a movie of the peeling step is shown in Supplementary Movie 1. A micrograph of the final structure is shown in Fig. 3i and reveals the successful formation of an aligned, tightly packed, concentric pattern of the two alternating metals. Three discrete gaps (G1, G2, G3) are formed between M1 and M2.

**Figure 4:**
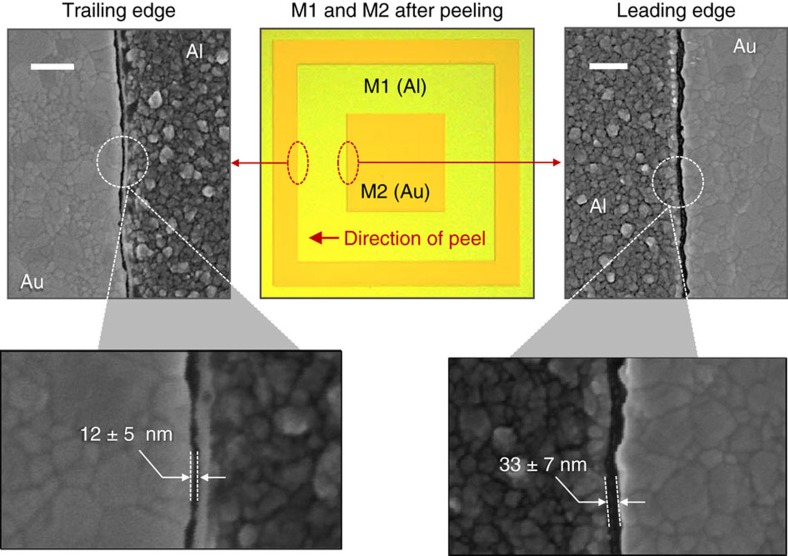
High-resolution imaging of concentric square electrodes. Micrograph of the concentric square electrode from Fig. 3i (shown centrally) with associated scanning electron micrographs of the Al/Au interface at the leading (right) and trailing (left) edges. Scale bars, 250 nm.

Figure 4 shows the micrograph from Fig. 3i, together with high-resolution scanning electron micrographs (HR-SEM)  of the Al/Au interfaces obtained at the leading (G1 junction) and trailing (G2 junction) edges. The images reveal the grain-like structure of the two metals and the formation of a tight intergranular boundary between them, along which the gold (M2) fractures during peeling. Size analysis of ~\n2 μm sections along the two interfaces indicated separations of 33±7 nm at the leading edge of the Al (M1) and 12±5 nm at the trailing edge (see Supplementary Fig. 6)—remarkably narrow for a simple mechanically induced process that involves no intricate alignment procedures. The gap widths nonetheless substantially exceed the few-nanometre length of the SAM molecules, which is expected to determine the ultimate resolution limit of the technique, suggesting considerable scope for further process optimization. The difference in gap widths at the two edges is consistent with the previously discussed difference in peeling forces—0.35 N at the leading edge versus 0.16 N at the trailing edge. The higher the peeling force the greater the risk that, in addition to sharp cracking along the SAM-induced nanogap, diffuse cracking will also take place through the formation of intergranular and transgranular microcracks in the region of the applied stress. Owing to the inherently distributed nature of such microcracks, a broadening of the final electrode spacing is expected to result. We note that the trailing edge histogram in Supplementary Fig. 6 includes a small number of gap spacings in the sub-4-nm range, indicating that the narrowest regions of the trailing edge nanogap are comparable in size to the SAM length. Significant reductions in the mean gap width may therefore be achievable using a yet weaker adhesive that provides lower (but sufficient) peeling forces at the M2/tape interface, while at the same time retaining sufficient mechanical rigidity to peel away in a single unbroken strip.

Importantly, despite the non-uniform edge profile of the prepatterned Al electrode, the two metals show a high degree of alignment along the length of the interface with a mean clearance of just 12 nm for the trailing edge. Hence, it is evident that nanoscale alignment can be achieved even with coarsely patterned electrodes (as for instance obtained by shadow mask deposition), thereby obviating the need for high-resolution e-beam or optical lithography at any point in the fabrication procedure. We note that the aspect ratio of the features in the image are of order ~\n100,000 (10^−3^ m/10^−8^ m)—a remarkably high value that compares favourably with previous reports in the literature where the term ‘very large aspect ratio’ has been used to describe values in excess of 1,000 (ref. 6).

Atomic force microscopy (AFM) of the gold surface (M2) before peeling indicates the principal reason for the remarkably narrow gap widths. A highly oriented macroscopically continuous grain boundary is visible directly above the edge of the aluminium in both the topography and phase images (Supplementary Figs 7a,c and 8) due to the presence of the conformally attached SAM. Hence, for fracturing to occur during the peeling step, it is necessary only to overcome the comparatively weak intergranular forces along the boundary (and then detach the Au from the SAM-coated Al)—a process that can yield a much sharper tear than fracturing of a randomly polycrystalline film. (Note, in the absence of a macroscopically continuous grain boundary, fracturing would instead need to occur during the peeling step via the formation of numerous intergranular and transgranular microcracks in the region of the applied stress, with the final fracture occurring when the cracks were sufficient in number and size to coalesce into a contiguous whole. Owing to the distributed nature of the constituent microcracks, the result would be a much rougher break that would not faithfully follow the boundaries of M1).

AFM images obtained after the peeling step (Supplementary Figs 7b,d and 9) confirmed the formation of a sharp interface between the Al and Au in agreement with the SEM images (although, due to the limited resolution of the AFM technique, a gap width cannot be inferred from the data).

### Fabrication and evaluation of nanogap photodiodes

**Figure 5:**
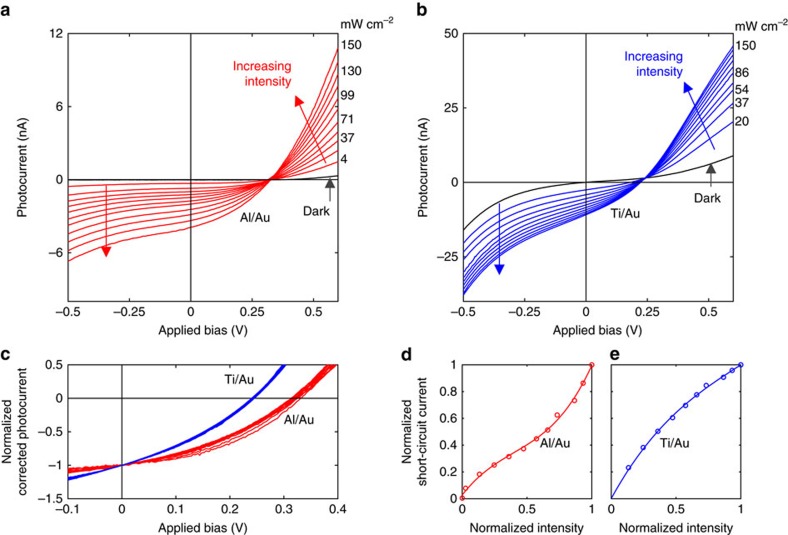
Electrical characterization of nanogap phototodiodes. (**a**,**b**) Current–Voltage (*I*–*V*) curves for Al/Au and Ti/Au nanogap photodiodes, formed by depositing a uniform composite layer of poly(3-hexylthiophene) (P3HT) and C_71_-butyric acid methyl ester (PCBM) across the surface of the substrate, see Supplementary Fig. 10. *I–V* curves were measured at multiple illumination intensities between 0 and 150 mW cm^−2^. (**c**) Normalized corrected photocurrent versus voltage curves for the Al/Au and Ti/Au nanogap photodiodes at illumination levels >20 mW cm^−2^, obtained by subtracting the dark current and dividing through by the magnitude of the short-circuit current. Consistent behaviour is observed at all illumination levels. (**d**,**e**) Normalized short-circuit current versus normalized light intensity for the Al/Au and Ti/Au nanogap photodiodes.

To evaluate the suitability of a-lith-patterned nanogap electrodes for practical device applications, Al/Au nanogap electrodes were fabricated (see Methods and Supplementary Fig. 10) and the (insulating) ODPA layer was removed by oxygen plasma ashing. Electrical contact was made to the two electrodes and the current (*I*) was measured as a function of the applied bias (*V*) over the range −1 to +1 V. The current remained below the 50 pA detection limit of the instrumentation across the full sweep (see Supplementary Fig. 11a), confirming the electrical isolation of the asymmetric electrodes and further indicating their physical separation. Next, coplanar organic photodiodes were fabricated by depositing a donor/acceptor blend of poly(3-hexylthiophene) (P3HT) and C_71_-butyric acid methyl ester (PCBM)^21^ uniformly over the nanogap electrodes (see Methods). The photovoltaic response of the resultant coplanar photodiodes was determined by carrying out repeated current–voltage sweeps from −0.6 to +0.6 V at multiple light intensities in the approximate range 0–150 mW cm^−2^. Well-behaved *I*–*V* curves were obtained in all cases (see Fig. 5a), with the photocurrent increasing monotonically in size with increasing light intensity. Normalized corrected photocurrent curves (obtained by subtracting the dark current from the illuminated *I*–*V* curves and dividing through by the short-circuit current) were nearly identical in shape at illumination levels >20 mW cm^−2^, with similar fill factors of 37±2% and open-circuit voltages of 0.32±0.01 V, indicating reproducible device behaviour at all illumination levels (see Fig. 5c). Plotting the short-circuit current versus incident light intensity yielded a super-linear response (see Fig. 5d), suggesting a moderate increase in the efficiency of free carrier generation at higher light intensities.

Lastly, to demonstrate the applicability of the a-lith method to other electrode materials, P3HT/PCBM photodiodes were fabricated using lightly oxidized titanium (M1) and Au (M2) as the nanogap electrodes. Ti/Au electrodes were first fabricated in a similar manner to the Al/Au electrodes, using ODPA as the SAM (see Methods for fabrication details and Supplementary Figs 12–14 for corresponding AFM and SEM images). Current–voltage sweeps carried out after removal of the ODPA by oxygen plasma ashing indicated excellent electrical isolation between the electrodes, with currents again remaining below the 50 pA detection limit of the instrumentation throughout the sweep (see Supplementary Fig. 11b). Following deposition of the P3HT:PCBM layer, the photodiodes exhibited similar *I*–*V* characteristics to the Al/Au photodiodes, albeit with a slightly higher dark current and higher short-circuit currents (see Fig. 5b). The normalized corrected photocurrent curves were again consistent in shape above 20 mW cm^−2^, with fill factors of 33±1% and open-circuit voltages of 0.24±0.005 V (see Fig. 5c). In contrast to the Al/Au devices, a plot of short-circuit current versus light intensity yielded a sublinear curve (see Fig. 5e), suggesting a reduction in the efficiency of free carrier generation with increasing light intensity. (Note, the insensitivity of the normalized corrected photocurrent curves to light intensity argues against changes in the rate of non-geminate bimolecular recombination being the cause of the non-linearity for either the Al/Au or the Ti/Au photodiodes^22^. This leaves changes in the efficiency of free carrier generation as the most probable cause.)

Notably, both sets of devices operated as photodiodes (as opposed to photoconductors) due to their use of dissimilar electrodes, with the Al/Au devices showing especially good rectification characteristics for such a narrow device width: |*I*(0.6)/*I*(−0.6)| ~\n15 under dark conditions. This contrasts with previously reported P3HT/PCBM nanogap devices using symmetric e-beam-patterned gold contacts with gap widths down to 50 nm, which exhibited non-rectifying device characteristics and operated as photoswitches, requiring the application of an external bias to detect light^23^. The requirement for asymmetric electrodes was previously discussed by Pang and co-workers^24^, who reported P3HT/PCBM nanogap photodiodes using titanium and reduced graphene oxide electrodes separated by a 500 nm gap. Despite the substantially lower gap width, the devices reported here compare favourably with their devices, showing a clear reproducible photovoltaic response in the absence of an applied bias.

## Discussion

The patterning resolution that can be achieved using a-lith compares favourably with the very few reports of aligned asymmetric nanogaps in the literature^4^,^5^, which have utilized e-beam lithography to achieve the requisite registration of the two metals. Using a combination of optical lift-off lithography, e-beam lithography, plasma-enhanced vapour deposition and chemical etching, Gao *et al.*^4^ reported an 11-step route to asymmetric electrodes capable of yielding well aligned nanogaps of Ti and Au down to 5 nm. Guillorn *et al.*^5^ meanwhile reported a sophisticated procedure, utilizing high-resolution e-beam lithography, two stages of reactive ion etching, thermal and electron gun physical vapour deposition of the metals and lift-off pattern transfer to achieve asymmetric electrode pairs of Au and Pt, Ti, Pd or Al with interelectrode spacings of 6 nm. A further technique for fabricating asymmetric nanogap electrodes was reported by Deshmukh *et al.*^11^, who used conventional e-beam lithography to first define point-like gold electrodes with a separation of ~\n250 nm, and then electrodeposited a second metal (Co or Cu) onto one of the electrodes to narrow the gap, thereby attaining a nanometre-sized electrode separation.

While a-lith-based patterning cannot yet match these methods in terms of the minimum achievable electrode spacing, it is remarkable that such a simple process—performed without costly equipment under ambient conditions on a timescale of seconds—can routinely yield electrodes that are only marginally less proximate. The method moreover may be applied over extended (>>1 mm) length-scales, for which e-beam methods are unsuited. Significantly, the SEM micrographs for the trailing edge nanogaps indicate the occurrence of sub-4-nm spacings at multiple points along the metallic nanogap, suggesting further reductions in the mean gap width should be achievable with process optimization. Improved methods for peeling away the upper metal layer in a more controlled manner are now under investigation, with a view to realizing the ultimate resolution limit of the a-lith method determined by the length of the SAM. The application of a-lith to the fabrication of densely packed arrays of two- and three-terminal nanogap electrodes and devices offers a promising route to the fabrication of nanoscale diodes and transistors that would be difficult to fabricate economically by any other method.

Finally we note that, while we have reported results for Au, Al and Ti metals, a-lith should prove applicable to a broad range of thin-film metals, oxide-coated metals and semiconducting metal oxides. The formation of dense, conformal, well-ordered coatings of alkyl-terminated SAMs has been reported for numerous metals and metal oxides, including Ag, Cu, Pd, Pt, Ni, Zn, Fe_*x*_O_*y*_, TiO_2_, ZrO_2_ and indium tin oxide^25^, so there is good reason to expect the interlayer method will be compatible with the majority of commonly used electrode materials provided a suitable SAM is available.

## Methods

### Deposition of Al(M1)/Au(M2) bilayer with or without ODPA

Thermally cross-linkable benzocyclobutene (BCB) polymer was deposited onto clean 2 × 2 cm glass substrates by spin casting the as-received material (Cyclotene 3022–46, Dow Chemicals) at 2,000 r.p.m. for 30 s in nitrogen. The films were preannealed on a hot plate in a nitrogen atmosphere (<10 p.p.m. O_2_) at 170 °C for 60 min to partially cross-link the BCB.

To prepare structures without ODPA, the substrates were loaded into a high vacuum (10^−6^ mbar) thermal evaporator and 40 nm layers of Al and Au were sequentially deposited at 1 Å s^−1^. To prepare structures with ODPA, the substrates were removed from the thermal evaporator after deposition of the Al layer, cleaned in an oxygen plasma (100 W, O_2_ flow rate: 3 ml min^−1^) for 10 min and then immersed in a 5 mM solution of ODPA in isopropanol (IPA) for 48 h. The films were then thermally annealed at 120 °C for 1 h in N_2_, before rinsing lightly in IPA to remove unbound/residual SAM molecules. The substrates were then returned to the thermal evaporator for deposition of the Au layer (40 nm, 1 Å s^−1^). Finally, all substrates were postannealed for 4 h at 200 °C in N_2_ to complete the cross-linking of the BCB.

### Deposition of Au(M1)/Al(M2) bilayer with or without ODT

To prepare structures without ODT, clean 2 × 2 cm glass substrates were loaded into a high vacuum (10^−6^ mbar) thermal evaporator and a thin (5–10 nm) layer of thermally evaporated Cr was deposited onto the glass at 1 Å s^−1^. 40 nm layers of Au and Al were then sequentially deposited at 1 Å s^−1^. To prepare structures with ODT, the substrates were removed from the thermal evaporator after deposition of the Au layer, cleaned in an oxygen plasma (100 W, O_2_ flow-rate: 3 ml min^−1^) for 10 min and then immersed in a 5 mM solution of ODT in IPA for 48 h. The films were then rinsed lightly in IPA to remove unbound/residual SAM molecules, before returning the substrates to the thermal evaporator for deposition of the Al layer (40 nm, 1 Å s^−1^).

### Fabrication of M1 line arrays with M2 coating

Fabrication was carried out using the procedures described above (with the SAM present), except the line array pattern was defined by evaporating M1 though a shadow mask. The spacings, widths and lengths of the line arrays were 4, 1.2 and 20 mm, respectively.

### Fabrication of concentric square Al/Au electrodes

Fabrication was carried out using the same general procedure used for the Al/Au line arrays (with ODPA present), except the Al layer (M1) was patterned photolithographically in a concentric ‘square-ring in square-ring’ geometry as follows: (1) S 1805 positive photoresist (Shipley Microposit) was deposited on top of the Al by spin coating the as-received solution at 4,000 r.p.m. for 40 s; (2) the resist was soft baked at 115 °C for 60 s; (3) it was then selectively exposed at 365 nm for 6 s (10 mW/cm^2^) via a chrome-plated quartz shadow mask, using a mask aligner (Karl Suss MA3); (4) the substrate was immersed in MF-319 developer (AZ electronic materials) for 90 s, rinsed and dried; (5) The Al was then etched by immersion in Al etchant (ANPE 80/5/5/10, Microchemicals) at 40 °C for ~\n30 s; (6) finally, the photoresist was removed by rinsing repeatedly in acetone and IPA.

### Peel test procedure for M1/M2 bilayers

Peel tests were carried out using the 90° peel test apparatus shown schematically in Supplementary Fig. 1. The substrate was firmly mounted on a light weight, low-friction horizontal stage. A length of polyvinyl chloride backed (PVC) insulation tape (AT7 PVC Insulation Tape, Advance Tapes) was used for the adhesive tape, and the final 2 cm was applied uniformly across the surface of M2. The other end of the tape was connected to a vertical stage via a digital force gauge (ELC-09S Tensile Load Cell, Xiamen Elane Electronics). The adhesive tape was pulled vertically from the substrate at a constant speed *V*, with the 90° geometry being maintained by means of a narrow horizontal bar oriented along the inner bend of the adhesive tape. The vertical force was recorded as a function of time using the strain gauge, and converted to a force versus displacement ‘peel-curve’ on the basis of the constant velocity.

### Peel test procedure for peeling M2 from M1 line arrays

Peel tests were carried out using the same 90° peel test procedure as used for using the same general procedure used for the bilayers, with either PVC insulation tape or adhesive glue (First Contact Red, Photonic Cleaning Technologies). The as-received adhesive glue was applied to the surface of M2 using the provided applicator brush.

### SEM

High-resolution SEM images (Fig. 4) were acquired using an FEI Nova Nano630 scanning electron microscope equipped with a field emission electron source and through-lens electron detectors. The electron-beam voltage and current conditions used for imaging were 2 kV and 8 pA, respectively. Standard resolution SEM images (Supplementary Figs 5 and 14) were acquired using a LEO 1525 Field Emission Scanning Electron Microscope at an operating voltage of 5 kV.

### Optical micrographs

High-magnification optical images were obtained using a Nikon LV100 optical microscope.

### AFM

Intermittent contact mode images were obtained using an Agilent (5500) AFM with silicon tips (Budget Sensors).

### AFM spectroscopy

Force–displacement curves were obtained in a dry nitrogen environment (<20 p.p.m. H_2_O) by static point AFM spectroscopy, using an Agilent (5500) AFM with NanoSensors PointProbe Plus FM-AFM cantilevers (force constant: 2.8 N m^−1^). For each film, curves were recorded at 10 separate locations, with 10 curves being recorded at each location. The adhesion force was taken to be the maximum of the force adhesion curve during probe retraction.

### Fabrication of Al/Au and Ti/Au nanogap photodiodes

Al/Au nanogap electrodes were prepared on a clean glass substrate using the same procedure as for the concentric square electrodes, omitting the BCB layer and using the mask shown in Supplementary Fig. 10. Ti/Au nanogap electrodes were prepared in the same way, using ODPA-coated Ti for M1, except a 2:1 solution of 30% aq. H_2_O_2_:NH_4_OH was used as the titanium etchant. The architecture of the patterned electrodes is shown schematically in Supplementary Fig. 10. After a-lith patterning of the nanogap electrodes, the ODPA was removed by 10 min of oxygen plasma ashing (100 W, O_2_ flow rate: 3 ml min^−1^). The active layer was spin coated (1,000 r.p.m. for 35 s, 2,000 r.p.m. for 7 s) onto the electrode from a 0.7:1 weight-ratio solution of P3HT (Sigma) and C_71_-PCBM (Solenne) in chlorobenzene. Devices were annealed in a dry nitrogen atmosphere at 150 °C for seven minutes before testing.

### Current–voltage (I–V) measurements

*I–V* measurements were obtained in a nitrogen atmosphere using a Keithley 4200 semiconductor parameter analyser. Forward bias corresponded to Au being biased positively with respect to Al or Ti. The nanogap devices were illuminated through the glass substrate by placing a 470 nm light-emitting diode (Osram OSLON SSL) in contact with the glass substrate, and the incident intensity was varied from 0 to ~\n150 mW cm^−2^.

## Author contributions

D.J.B. conceived the interlayer lithography method for the fabrication of intimately spaced aligned electrodes. T.D.A. conceived its use for creating asymmetric metal electrode nanogaps and their use in coplanar opto/electronic devices. D.J.B., J.S., T.D.A. and J.C.dM. designed the experiments, analysed the experimental data and wrote the manuscript. D.J.B. carried out the experiments using Al/Au electrodes, with J.S. and T.D.A. extending the patterning procedure to Ti/Au electrodes. M.M., L.K.J. and A.A. conducted the electron microscopy measurements and provided feedback on the manuscript.

## Additional information

**How to cite this article:** Beesley, D. J. *et al.* Sub-15-nm patterning of asymmetric metal electrodes and devices by adhesion lithography. *Nat. Commun.* 5:3933 doi: 10.1038/ncomms4933 (2014).

## Supplementary Material

Supplementary Information Supplementary Figures 1-14, Supplementary Note 1 and Supplementary References

Supplementary Movie 1 Movie showing the peeling stage of adhesion lithography, in which the second metal M2 is detached from the regions directly above M1, leaving the two metals side by side on the substrate in a complementary arrangement.
