# Supplementary material for: Sub-15-nm patterning of asymmetric metal electrodes and devices by adhesion lithography
Source: Nat Commun. 2014 May 27;5:3933. doi: 10.1038/ncomms4933 (PMC4050269; doi:10.1038/ncomms4933)
Supplement: Supplementary Information — Supplementary Figures 1-14, Supplementary Note 1 and Supplementary References [file ncomms4933-s1.pdf]

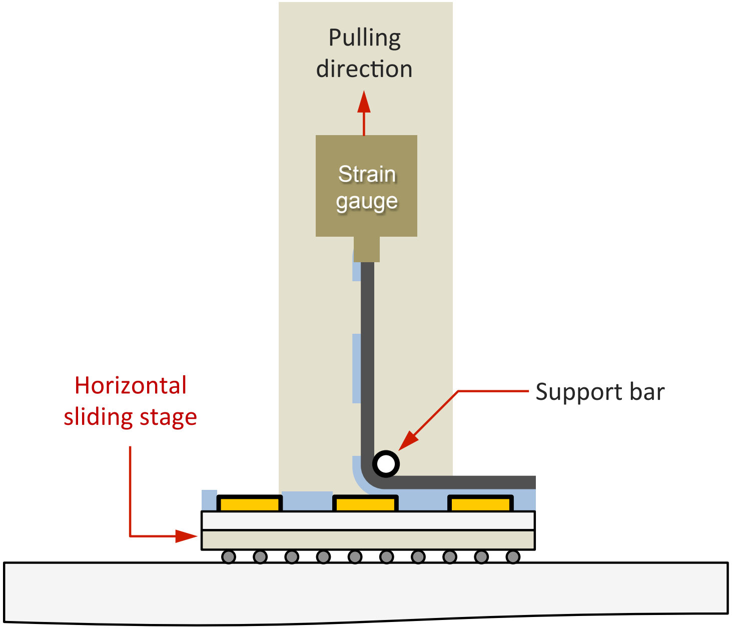

Supplementary Figure 1 | **Schematic of peel tester.** Diagram illustrating the geometry of a 90° peel test, using a ribbon of adhesive tape as a flexible adherent. The substrate is firmly attached to a light weight, low friction travelling stage that moves horizontally as the adhesive tape is pulled vertically from the substrate at a constant speed V. The 90° geometry is maintained by means of a narrow horizontal bar oriented along the inner bend of the adhesive tape. The vertical force is recorded as a function of time using a strain gauge, and converted to a force *versus* displacement "peel-curve" on the basis of the constant velocity.

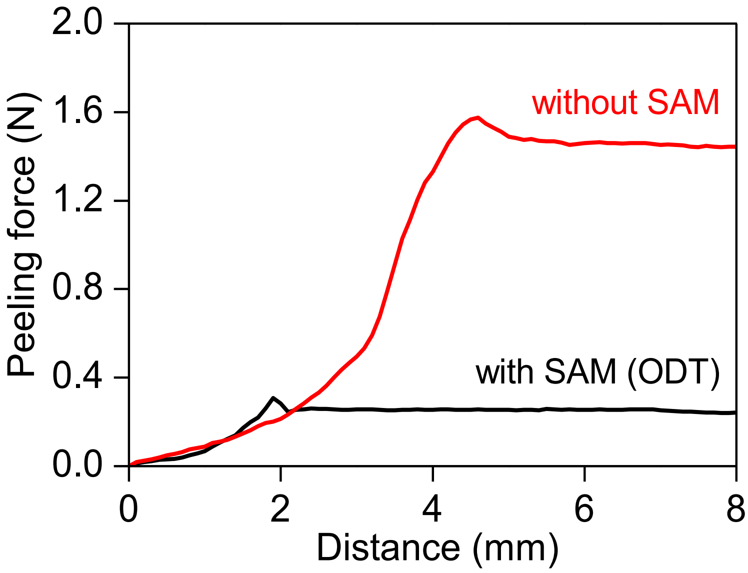

Supplementary Figure 2 | Peel test measurements for Al on Au. Peel-curves for a layer of aluminium on top of a uniform layer of gold, with and without an intervening SAM layer of octadecanethiol (ODT); curves were obtained using adhesive tape. The initial shallow response at low displacements corresponds to elimination of slack in the adhesive tape. This is followed by a steepening of the peel curve due to increased energy dissipation in the adhesive tape (see Supplementary Note 1). Without ODT the applied force peaks at a high value of ~1.6 N, before decreasing to a slightly lower steady-state value of ~1.45 N. With ODT the applied force peaks at a low value of ~0.3 N, before falling to a steady-state value of ~0.25 N.

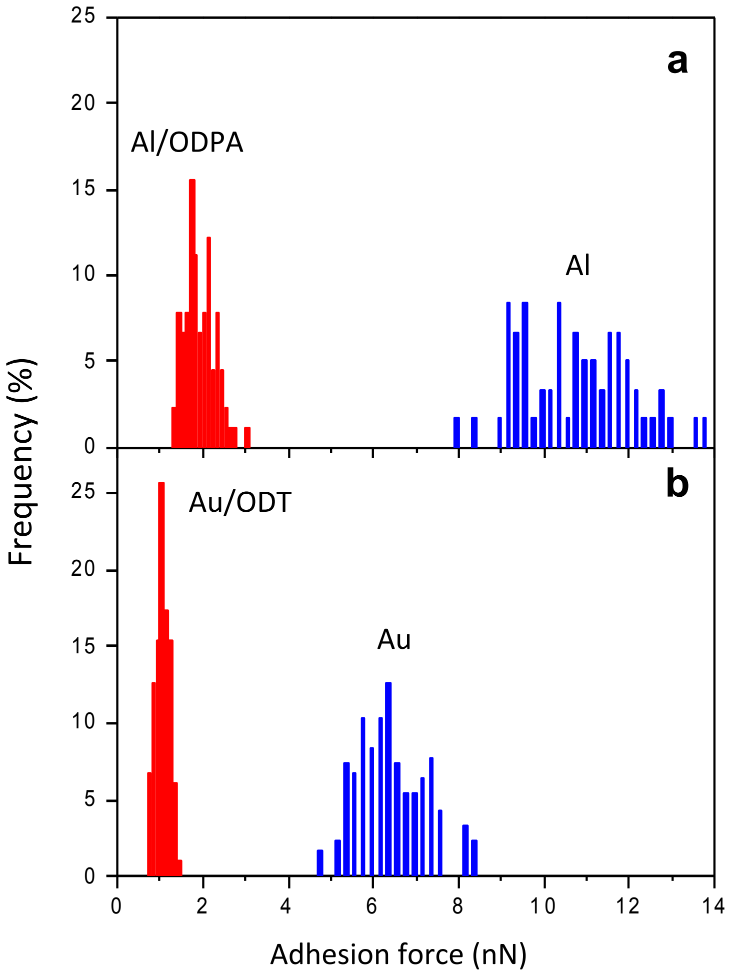

Supplementary Figure 3 | Influence of SAM on AFM adhesion-force measurements. Adhesion force histograms for metal films with and without a self-assembled monolayer. The local adhesion forces were determined by AFM force spectroscopy, taking the adhesion force to be the maximum of the force-displacement curve during tip retraction. In the case of Al (**a**) the mean adhesion force was reduced from ~11 nN without octadecylphosphonic acid (ODPA) to ~2 nN with, while for Au (**b**) it was reduced from ~6 nN without ODT to ~1 nN with, broadly consistent with the macroscopic results obtained by peel testing. In both cases a significant reduction in the spread of the adhesion forces was observed after application of the self-assembled monolayer, indicating a significant improvement in the uniformity of the surface.

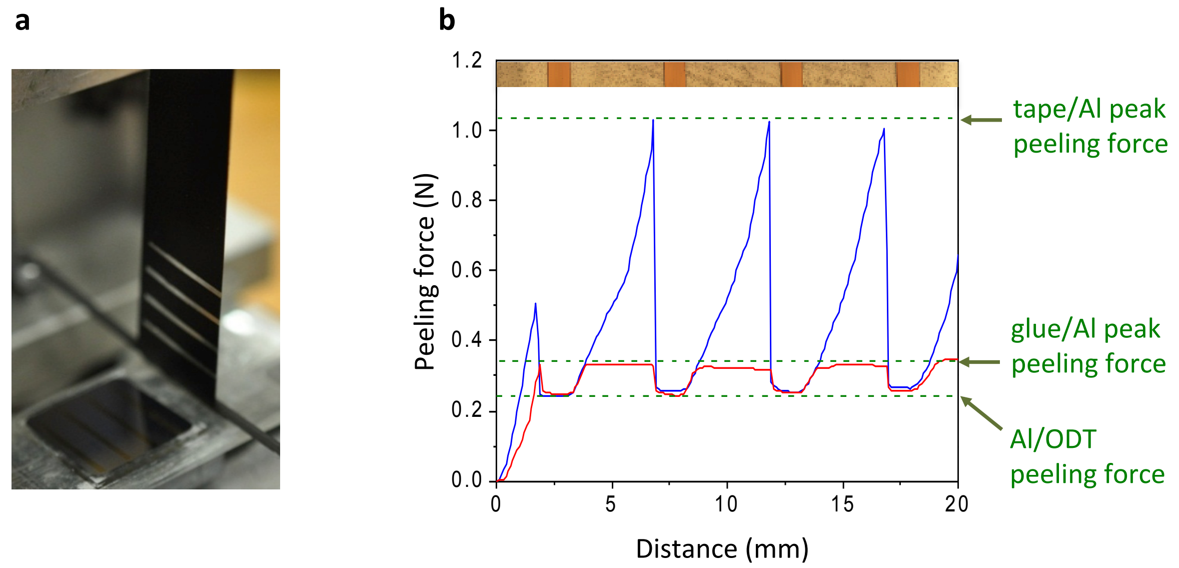

Supplementary Figure 4 | Peeling of Al from ODT-coated Au line-array. (**a**) Photograph showing tape after peeling Al from ODT-coated Au line arrays, with removed Al visible on the tape. (**b**) Peel curves for Al films on top of ODT-coated Au line arrays, using insulation tape or solution-coated glue as the adhesive layer. In the case of insulation tape a roughly linear increase in the peeling force from ~0.25 N to ~1 N is observed as the peel-edge passes across the (Au-free) regions between lines (due to an increase in curvature at the peel root), followed by a rapid decrease back to ~0.25 N as the peel-edge passes onto the ODT-coated Au. In the case of solution-coated glue a similar value of the peeling force is observed when the peel edge is located above the Au lines, but the behaviour in the region between lines is notably different, with the peeling force stabilising rapidly to ~0.35 N. The smaller peeling force differential between the Au and Au-free regions obtained using the glue is beneficial for achieving controlled peeling. The differing profiles of the peel curves are discussed in Supplementary Note 1. Inset shows a photograph of the final patterned structure.

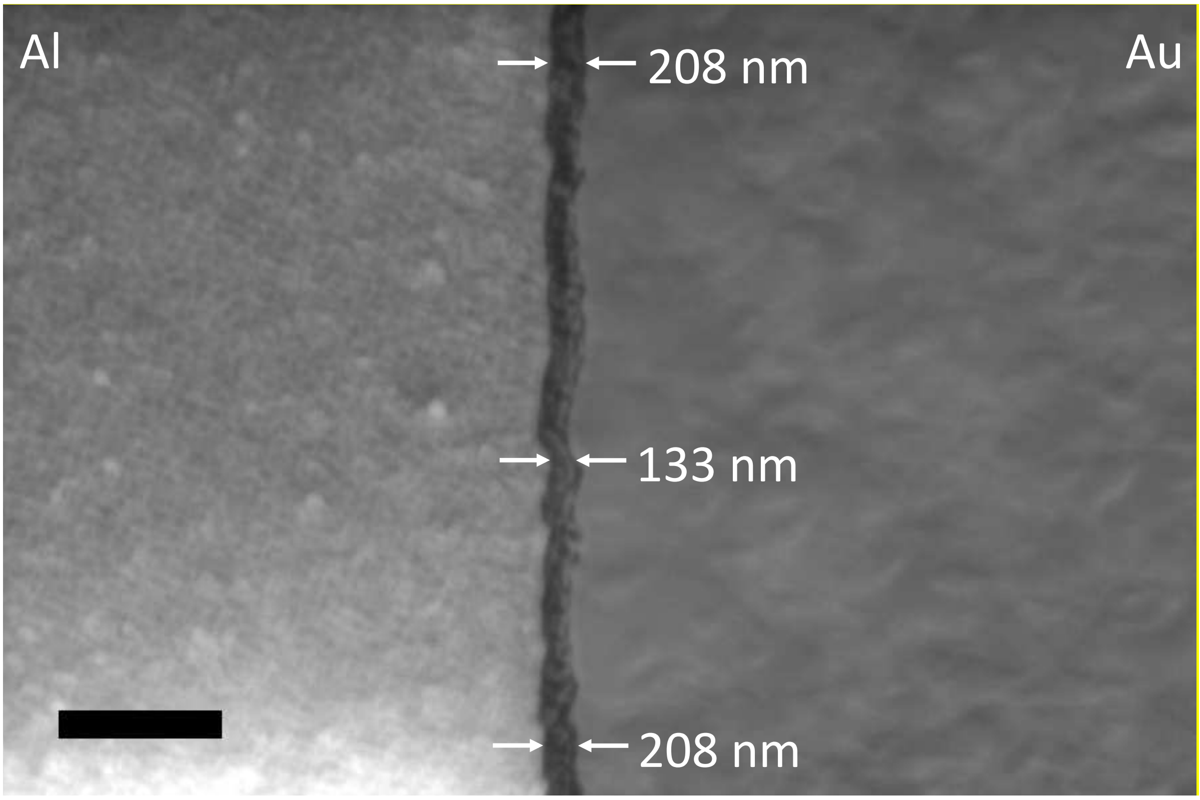

Supplementary Figure 5 | SEM micrograph of Al/Au electrode peeled by tape. The electrode was fabricated using ODPA-coated Al as M1 and Au as M2. Scale bar is 1 μm. (Note, all nanogap electrodes are specified in the form M1/M2, hence Al/Au implies M1 = Al and M2 = Au).

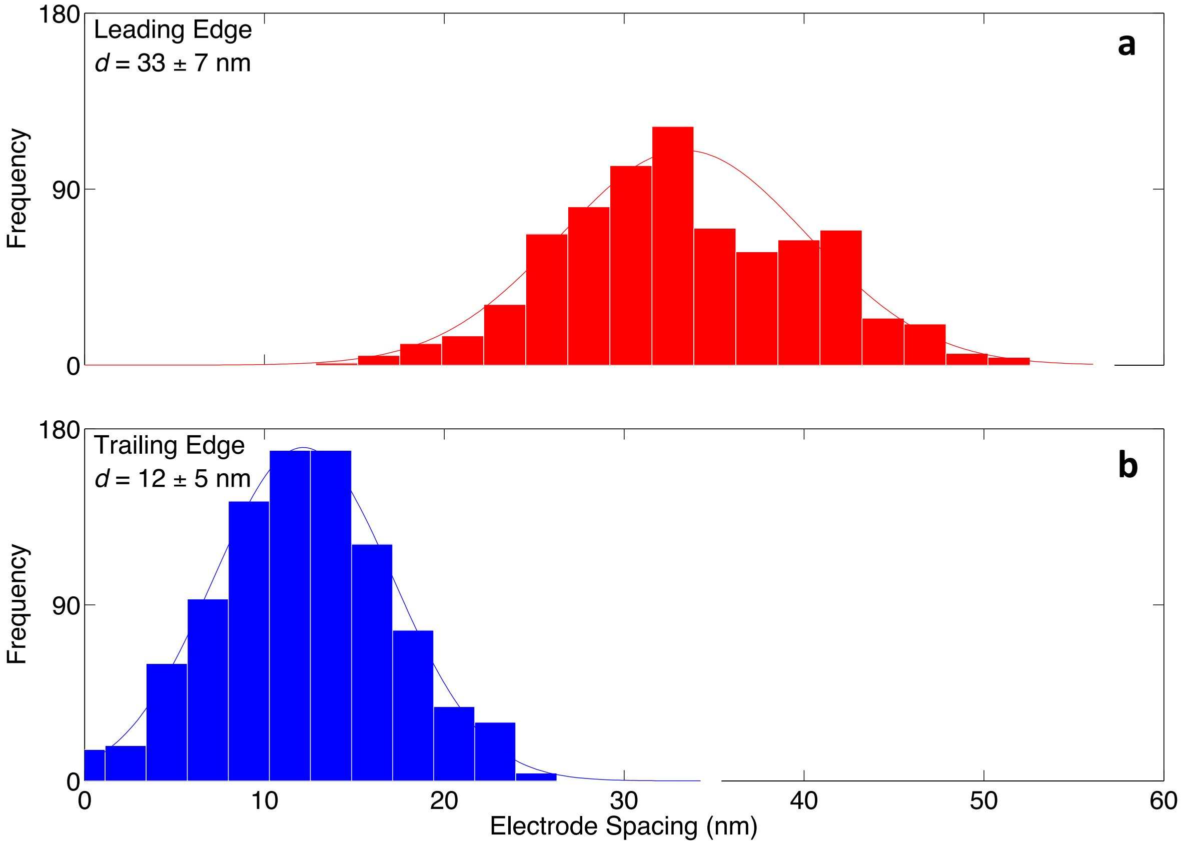

Supplementary Figure 6 | Histograms of measured nanogap spacings for concentric-square Al/Au electrodes. Data were obtained at ~2.3 nm intervals along ~2 μm measurement lines at the leading- and trailing- edges denoted in Figure 4. Peeling at the leading edge (**a**) yielded a gap with spacing 33 ± 7 nm (±1 SD), whereas peeling at the trailing edge (**b**) yielded a tighter gap with spacing 12 ± 5 nm.

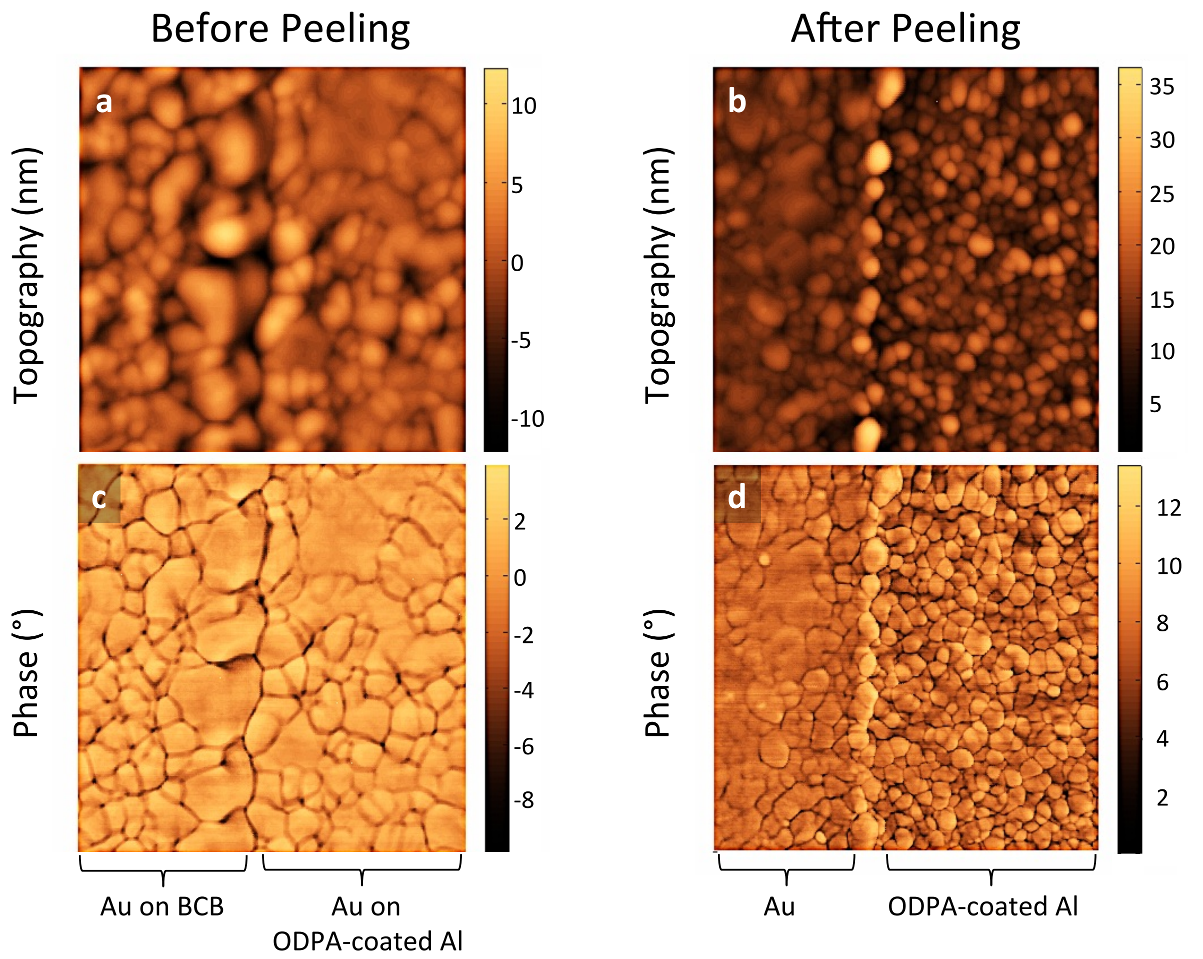

Supplementary Figure 7 | AFM imaging of Al/Au nanogap electrodes. AFM topography and phase images of the pre-peeled (**a**, **c**) and post-peeled (**b**, **d**) concentric-square-electrode from Figure 4, obtained at a trailing edge. Prior to peeling the structure comprises a uniform layer of Au (M2) on top of patterned ODPA-coated Al (M1), with a clear macroscopically continuous grain boundary in the Au running along the edge of the covered Al. After peeling the two metals sit side-by-side on top of the substrate and form a tight intergranular interface with one another.

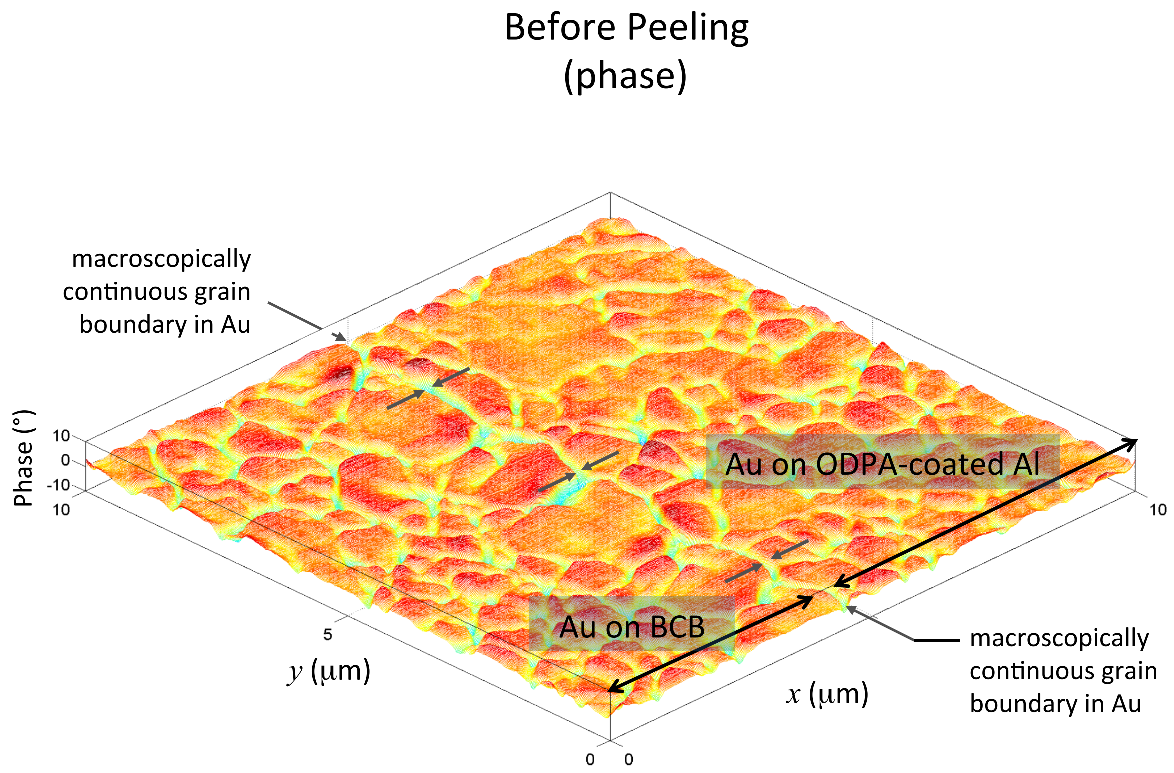

Supplementary Figure 8 | Pre-peeling phase plot of Al/Au nanogap electrode. Three dimensional surface plot of the AFM phase image from Supplementary Figure 7c, showing a macroscopically continuous grain boundary in the Au surface at $x \approx 5$ μm.

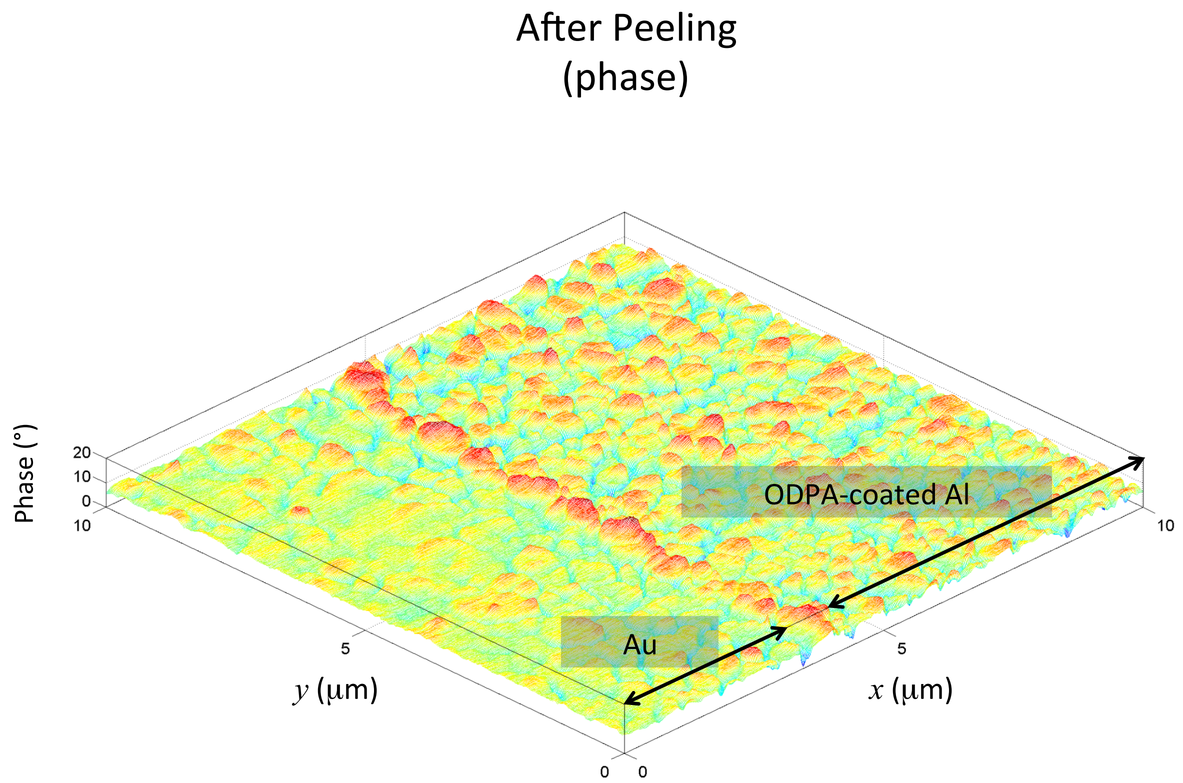

Supplementary Figure 9 | Post-peeling phase plot of Al/Au nanogap electrode. Three dimensional surface plot of the AFM phase image from Supplementary Figure 7d, showing the two metals sitting in close proximity side-by-side on the substrate.

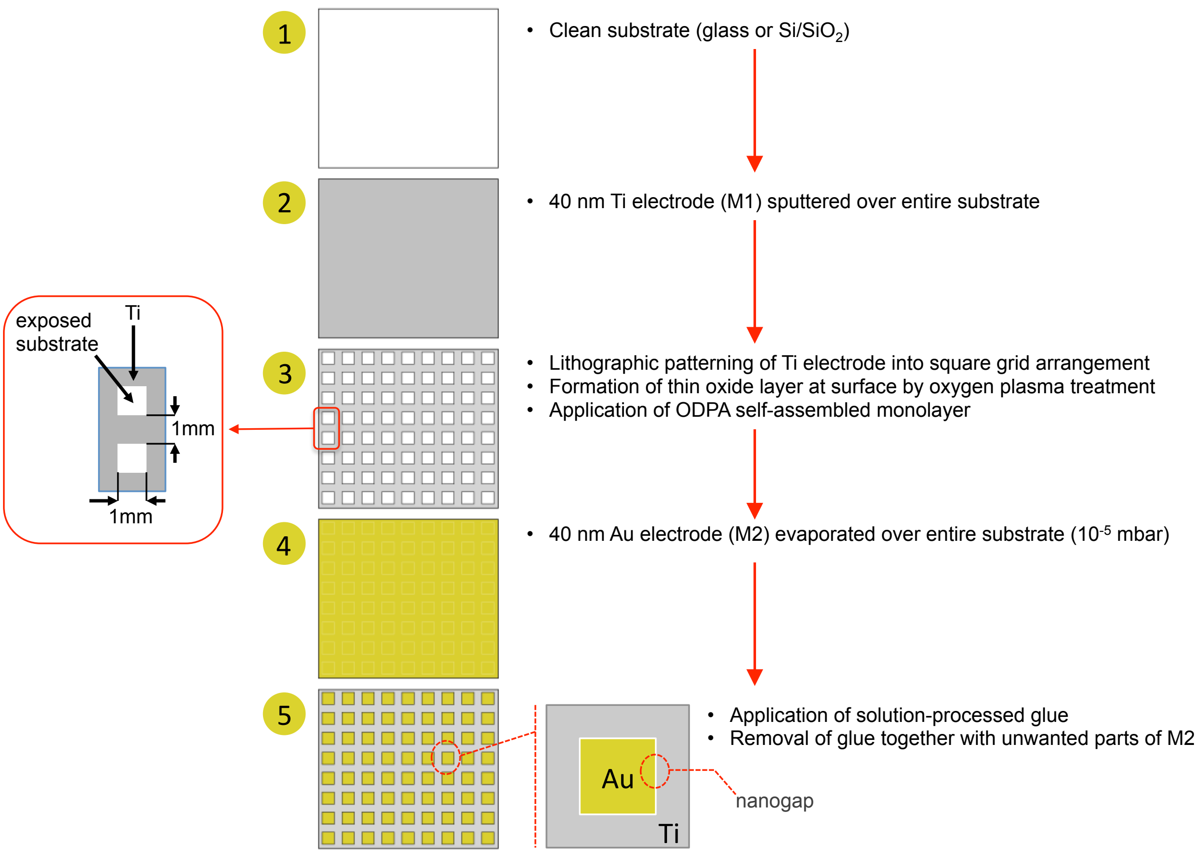

Supplementary Figure 10 | Key steps in the fabrication of a Ti/Au electrode array. The electrodes were converted into photodiodes by removing the self-assembled monolayer and depositing a uniform composite layer of P3HT/PCBM across the electrodes. The same mask design and coating procedure was used to fabricate the Al/Au photodiodes. See "*Fabrication of Al/Au and Ti/Au nanogap photodiodes*" in Methods for further details.

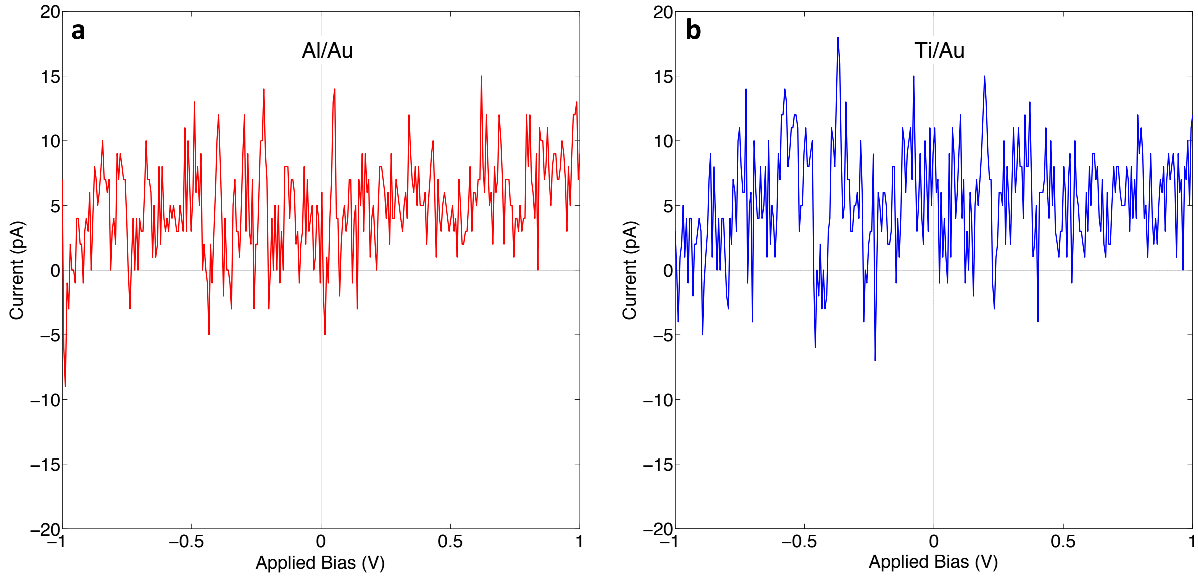

Supplementary Figure 11 | Current-voltage measurements for uncoated nanogap electrodes. Current voltage curves for uncoated Al/Au (**a**) and Ti/Au (**b**) nanogap electrodes fabricated according to Supplementary Figure 10. The measurements were made after removal of the ODPA self-assembled monolayer by plasma ashing. The negligible currents indicate excellent electrical isolation between the two electrodes.

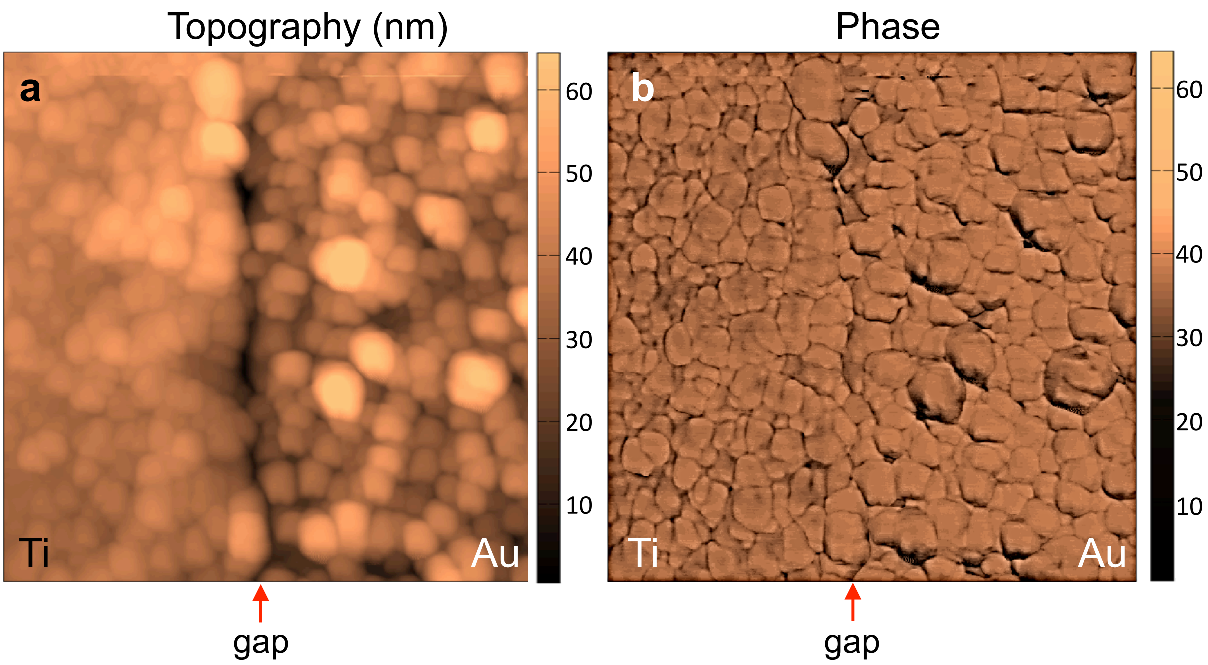

Supplementary Figure 12 | AFM imaging of Ti/Au nanogap electrode. AFM topography (**a**) and phase (**b**) images of a post-peeled Ti/Au trailing-edge nanogap electrode fabricated according to Supplementary Figure 10. The two metals sit side-by-side on top of the substrate and form a tight intergranular interface with one another.

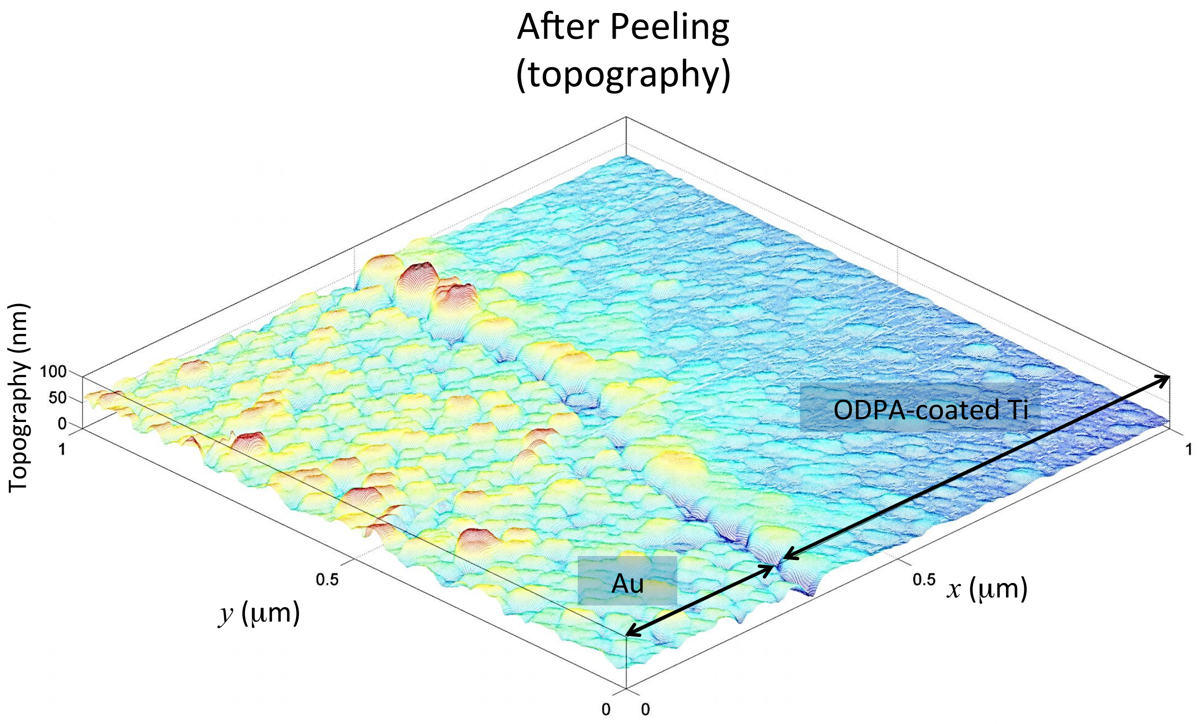

Supplementary Figure 13 | Post-peeling topography plot of Ti/Au nanogap electrode. Three dimensional surface plot showing the AFM topography of a Ti/Au trailing-edge nanogap electrode, with the two metals sitting in close proximity side-by-side on the substrate.

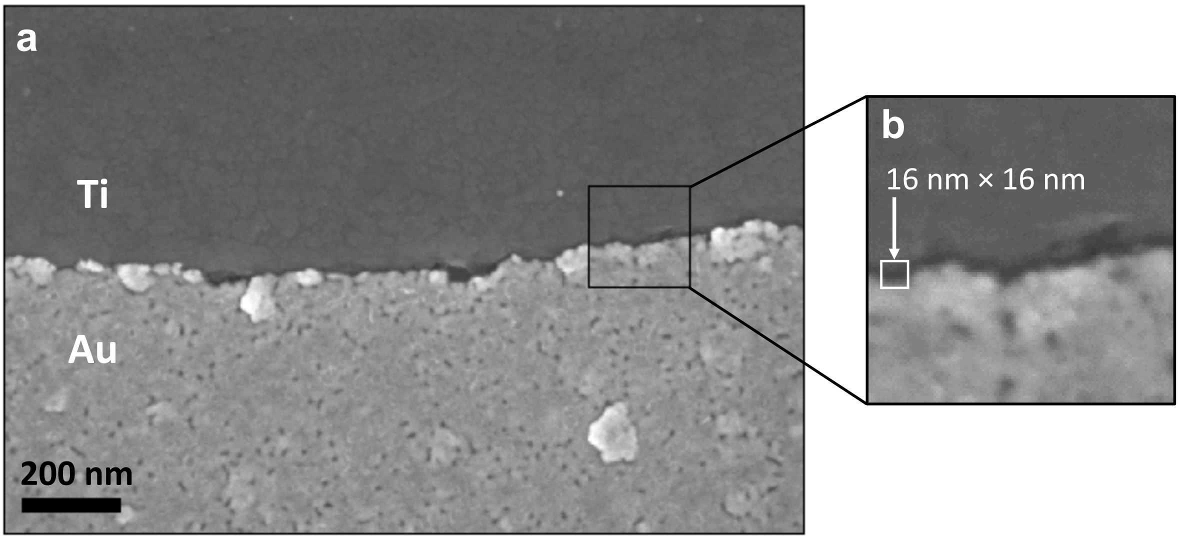

Supplementary Figure 14 | SEM imaging of a Ti/Au nanogap electrode. (**a**) SEM micrograph of a Ti/Au trailing-edge nanogap electrode fabricated according to Supplementary Figure 10 after peeling of the Au layer. (**b**) Expanded 200 nm x 200 nm section of the micrograph. The 16 nm × 16 nm white square encloses a small section of the nanogap.

Supplementary Note 1 - Further discussion of peel curve profiles

The peel curves in Figure 2c and Supplementary Figure 2 show an initial transient phase in which the peel force increases from a low initial value to a significantly higher steady-state value. In considering this behavior, we note that the peel force is not a direct measure of adhesion strength but instead corresponds to the force required to separate the two interfaces *plus* the force required to plastically deform the adhesive layer by bending. [1,2] This latter force, which is dependent on the configuration (shape) of the peeled adhesive, changes constantly throughout the transient phase. At the onset of peeling, the curvature of the adhesive close to the peel root is low, implying only weak energy dissipation due to plastic deformation. By the time steady-state is reached, however, the curvature is substantially higher and a much higher force is therefore required to induce plastic deformation. [2]

Analytical [1] and finite element [2] modeling show that, for a given adherent, the steady-state peel force is approximately proportional to the adhesion strength (with the steady-state being reached more rapidly in the case of low adhesion strength). Hence, although all four peel curves in Figure 2c and Supplementary Figure 2 exhibit a transient phase during which the peel force grows in strength, the steady-state peel force is significantly lower (and is reached sooner) with the SAM than without.

Referring now to the peel curves in Figures 2f and Supplementary Figure 4, it is straightforward to rationalise the different ways in which the two adhesives peel away from the line arrays. Each time the peel-edge moves beyond the trailing edge of M1 (where adhesion is weak) and traverses the SAM-free region of the substrate (where adhesion is strong), a monotonic increase in the applied force is observed in agreement with the transient-phase behaviour of Figure 2c and Supplementary Figure 2. In the case of the tape, it has not yet reached its steady-state configuration by the time it reaches the leading edge of M1, and hence no plateauing of the applied force is observed. On passing beyond the leading edge of M1 the applied force falls abruptly to a lower value, which corresponds to the steady-state force for delamination at the SAM/M2 interface: 0.16 N for ODPA/Au and 0.25 N for ODT/Al. The high-force-to-low-force transition occurs abruptly (i.e. the slope of the peel curve is almost vertical) because the tape is able to slacken immediately without further peeling taking place. The low-force-to-high-force transition, however, occurs gradually because the peeling of the

tape and the change in its configuration take place concurrently. Hence the overall peel curve acquires a saw-tooth-like appearance with regions of gradual increase followed by step-like drops.

The same general behaviour is observed with the solution-processed glue. However, since the adhesion strength at the M2/glue interface is significantly weaker than the adhesion strength at the M2/tape interface, steady-state is reached rapidly while the peel edge is still traversing the SAM-free zone and so the peel curve plateaus before the peel edge has reached the leading edge of M1. On passing beyond the leading edge of M1, the applied force again drops abruptly, falling to the steady-state peel force for delamination at the SAM/M2 interface: 0.16 N for ODPA/Au and 0.25 N for ODT/Al. The fact that the peel force in the SAM-coated zone is the same for the tape as for the glue indicates negligible plastic deformation of the adherents when delamination occurs at the SAM/M2 interface. In this case we are therefore directly observing the adhesive strength of the interface.

Supplementary References

1 Kim, K. S. & Kim, J. Elasto-plastic analysis of the peel test for thin-film adhesion. Journal of Engineering Materials and Technology-Transactions of the Asme 110, 266-273 (1988).

2 Cui, J., Wang, R., Sinclair, A. N. & Spelt, J. K. A calibrated finite element model of adhesive peeling. International Journal of Adhesion and Adhesives 23, 199-206, doi:10.1016/s0143-7496(03)00012-5 (2003).
